# Evolving Significance and Future Relevance of Anti-Angiogenic Activity of mTOR Inhibitors in Cancer Therapy

**DOI:** 10.3390/cancers9110152

**Published:** 2017-11-01

**Authors:** Seraina Faes, Tania Santoro, Nicolas Demartines, Olivier Dormond

**Affiliations:** Department of Visceral Surgery, Lausanne University Hospital, Pavillon 4, avenue de Beaumont, 1011 Lausanne, Switzerland; seraina.faes@chuv.ch (S.F.); tania.santoro@chuv.ch (T.S.); demartines@chuv.ch (N.D.)

**Keywords:** mTOR, tumor angiogenesis, rapalogs, cancer, endothelial cell

## Abstract

mTOR inhibitors have demonstrated remarkable anti-tumor activity in experimental models, mainly by reducing cancer cell growth and tumor angiogenesis. Their use in cancer patients as monotherapy has, however, generated only limited benefits, increasing median overall survival by only a few months. Likewise, in other targeted therapies, cancer cells develop resistance mechanisms to overcome mTOR inhibition. Hence, novel therapeutic strategies have to be designed to increase the efficacy of mTOR inhibitors in cancer. In this review, we discuss the present and future relevance of mTOR inhibitors in cancer therapy by focusing on their effects on tumor angiogenesis.

## 1. Introduction 

The mechanistic target of rapamycin (mTOR) is a serine/threonine kinase that exerts its effect by forming an integral part of two structurally and functionally distinct protein complexes, named mTOR complex 1 (mTORC1) and mTOR complex 2 (mTORC2) [1,2]. mTORC1 coordinates cell growth in favorable extracellular conditions by stimulating protein, lipid, and nucleotide synthesis, and by inhibiting autophagy [3]. mTORC2 is primarily activated by growth factors, and stimulates cell proliferation and survival by activating members of the group of AGC protein kinases, such as AKT [4,5]. The mTOR signaling pathway is frequently overactivated in cancer cells, either by mutations of upstream components of the pathway or by mutations of mTOR itself [6,7]. In addition, the mTOR pathway participates in tumor angiogenesis [8]. Hence, targeting mTOR has the potential to slow down tumor progression. 

**Figure 1:**
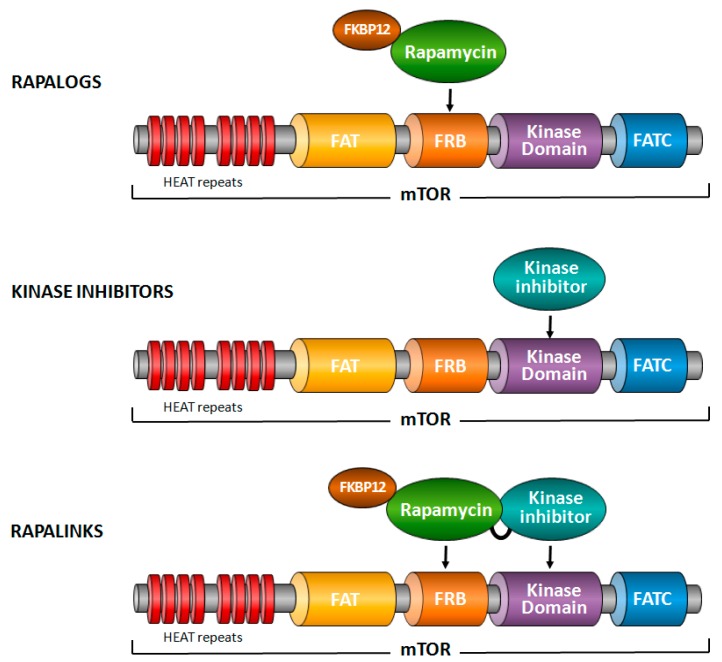
mTOR inhibitors. Three different types of mTOR inhibitors have been developed. Rapalogs, such as rapamycin, bind together with FKBP12 to the FRB domain of mTOR and block some functions of mTORC1. Kinase inhibitors of mTOR bind to the kinase domain of mTOR and block both mTORC1 and mTORC2. Rapalinks are composed of rapamycin cross-linked to a kinase inhibitor of mTOR.

Three different types of chemical inhibitors of mTOR have been tested in various cancer models (Figure 1) [9,10]. Firstly, rapamycin and its analogs—generally termed rapalogs—which bind, together with FKBP12 (FK506-binding 12 kDa protein), to the FRB domain of mTOR and exert a specific inhibition of mTORC1. This inhibition is, however, incomplete, as some epitopes phosphorylated by mTORC1 are resistant to rapalogs [11]. In addition, rapalogs do not directly block mTORC2, but might inhibit it in certain cell types following prolonged treatment [12]. Secondly, ATP-competitive inhibitors of mTOR that target the kinase domain of mTOR [13]. In contrast to rapalogs, these inhibitors completely block the activity of mTORC1 and mTORC2. Some of these inhibitors block PI3K as well, and are then named dual PI3K/mTOR inhibitors. Thirdly, and more recently, a compound composed of rapamycin cross-linked with a kinase inhibitor of mTOR has been generated, aiming to overcome resistance mutations to rapalogs or kinase inhibitors of mTOR [14].

In 1981, the anti-cancer activity of rapamycin was reported in various cancer cell lines [15]. Since then, numerous pre-clinical studies have confirmed that blocking mTOR impairs tumor progression [16,17]. Decreased cancer cell proliferation and reduced tumor angiogenesis are frequently associated with this effect. mTOR inhibitors have also demonstrated anti-cancer activity in patients, albeit limited, increasing median overall survival by a few months [18,19,20]. Hence, mTOR inhibitors used as monotherapy do not provide the expected anti-cancer efficacy. Several resistance mechanisms that dampen the effects of mTOR inhibitors have been identified [21]. The place of mTOR inhibitors in cancer needs, therefore, to be reconsidered, and novel therapeutic strategies based on mTOR inhibition have to be established. In this review, we discuss and speculate about the future use of therapies that target mTOR in cancer by focusing mainly on their effects on tumor endothelial cells.

## 2. mTOR Inhibitors and Tumor Angiogenesis

Blood supply in tumors is primarily established by formation of new blood vessels from pre-existing vascular networks in a process called angiogenesis [22]. A variety of cells, including tumor and tumor-associated stromal cells, participate in this process, in part by secreting growth factors and cytokines that stimulate tumor endothelial cells [23]. Tumor hypoxia is a key driver of angiogenesis, as hypoxic tumor cells secrete vascular endothelial growth factor (VEGF), which represents a major angiogenic factor [24,25]. Starving cancers by blocking tumor angiogenesis has been developed extensively over the past two decades [26]. Based hereon, therapeutic strategies targeting VEGF have shown clinical benefits that are, however, frequently not long-lasting [27].

**Figure 2:**
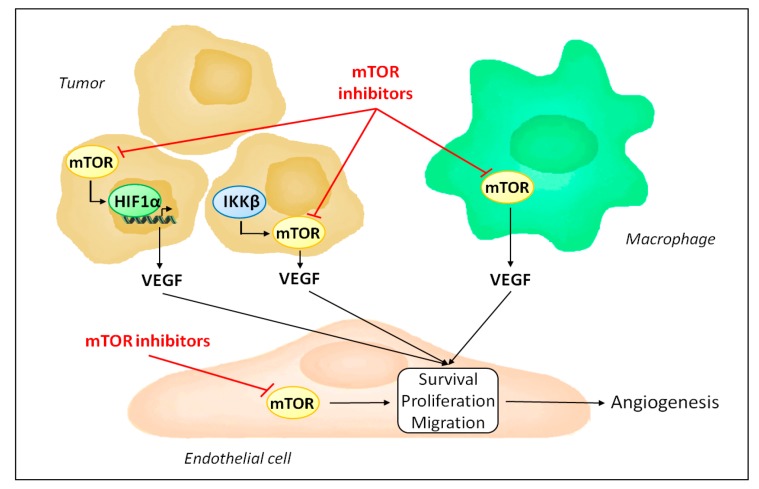
Mechanisms by which mTOR inhibitors affect tumor angiogenesis. mTOR inhibitors reduce endothelial cell proliferation, survival and migration by blocking endothelial mTOR. In addition, they decrease VEGF production, as mTORC1 is required to stabilize HIF1α during the hypoxic tumor response and is activated by IKKβ in inflammation-mediated angiogenesis. Finally, mTOR inhibitors induce tumor associated macrophage polarization to an anti-angiogenic phenotype.

Similarly to anti-VEGF treatments, mTOR inhibitors display anti-angiogenic properties [8]. This observation is supported by several in vitro and in vivo studies (Figure 2). In vitro, rapamycin was shown to markedly reduce spontaneous and growth factor-mediated endothelial cell proliferation [28,29,30,31]. This is associated with decreased cyclin D1 expression and a consequent reduction of S-phase entry by endothelial cells [31,32]. MicroRNAs (miRs) also influence the anti-proliferative effects of mTOR inhibitors in endothelial cells. Rapamycin increases expression of miR-21 in endothelial cells, and a downregulation of miR-21 abolishes the anti-proliferative effects of rapamycin [33]. Importantly, inhibition of mTOR by rapamycin also inhibits hypoxia-mediated endothelial cell proliferation [34].

Besides endothelial cell proliferation, rapamycin further influences other cell functions relevant to tumor angiogenesis. For instance, growth factor stimulated endothelial cell sprout formation in rat or mouse aorta is reduced by rapamycin [34]. In addition, rapamycin decreases endothelial cell survival [35,36,37]. In serum and growth-factor deprived conditions, VEGF-induced endothelial cell survival is inhibited by rapamycin as evidenced by flow cytometry analysis of annexin stainings or cell cycle profile of endothelial cells [35,37]. Pro-apoptotic effects of rapamycin in endothelial cells are mediated by its ability to inhibit mTORC2 activity and consequently AKT phosphorylation and activation [37]. Rapamycin also reduces endothelial cell migration [36,37,38]. Molecular mechanisms involved in this matter include increased expression of the cyclin-dependent kinase inhibitor p27, as well as miR-21 by rapamycin [33,39]. Finally, rapamycin further reduces the ability of endothelial cells to form tubular structures in vitro [40,41].

In addition to rapalogs, the effects of kinase inhibitors of mTOR on endothelial cell proliferation, survival, migration, and tube formation have been tested. These inhibitors possess similar but stronger activities than rapalogs on endothelial cells in vitro [42]. 

The anti-angiogenic properties of mTOR inhibitors have also been illustrated in various cancer models in vivo. For instance, rapamycin decreases angiogenesis in dorsal skin fold chambers transplanted with tumor cells, and in tumor xeno- and allografts [40]. The anti-angiogenic potential of the rapalog CCI-779 was demonstrated in the matrigel plug assay, where CCI-779 inhibited VEGF-stimulated vessel formation [43]. The latter further reduced microvessel density in two different rhabdomyosarcoma xenografts [44]. Reduced vessel density in these models was associated with decreased level of HIF-1 (hypoxia-inducible factor 1) and VEGF, confirming the role of mTOR in hypoxic tumor response. Besides tumor xeno- and allografts, rapalogs also demonstrated anti-angiogenic efficacy in a transgenic mouse model characterized by the development of ovarian serous adenocarcinomas [45]. In addition, rapalogs further decreased vascular density in patient-derived hepatocellular carcinoma xenografts [46]. Interestingly, the rapalog RAD001 reduced the growth of tumor xenografts generated from cancer cell lines that are either sensitive or insensitive to RAD001 in vitro. In either case, RAD001 reduced the number of tumor blood vessels in tumor xenografts, highlighting the anti-angiogenic effect of rapalogs as a major mechanism to decrease tumor xenograft growth [47]. The amount of intra-tumoral VEGF was also reduced by RAD001. Besides histological analysis, the anti-angiogenic activity of rapamycin has been evidenced by magnetic resonance imaging [48].

Finally, and more importantly, the anti-angiogenic effect of rapalogs has been reported in tumor patients [49]. Lymph node biopsies retrieved before and after treatment of a patient suffering from mantle cell lymphoma with CCI-779 revealed a decrease of tumor blood-vessel density. 

Similarly to rapalogs, several studies have demonstrated anti-angiogenic effects of ATP-competitive inhibitors of mTOR. The dual PI3K/mTOR inhibitor NVP-BEZ235 showed anti-angiogenic activity in tumor mouse models of breast and renal cell cancers and glioma [50,51,52]. NVP-BEZ235 also reduced intra-tumoral levels of VEGF [52]. The anti-angiogenic effects of NVP-BEZ235 were more pronounced than with rapalogs [42,51,53]. Similarly, selective kinase inhibitors of mTOR reduced vessel density in various models [42,54,55]. For instance, the mTORC1/mTORC2 kinase inhibitor OXA-01 decreased tumor blood vessels and intra-tumoral levels of VEGF more potently than rapamycin [55]. Analogous findings were reported for PP242 [56].

Despite a clear anti-angiogenic activity of mTOR inhibitors in tumor mouse models, few studies have investigated their effects on tumor endothelial cells in vivo. Nevertheless, it was reported that rapamycin increased tumor endothelial cell apoptosis in orthotopic pancreatic tumors using terminal deoxynucleotidyl transferase nick end labeling (TUNEL) and CD31 double staining [35]. This was associated with damaged vessels containing thromboses. Formation of vessel thrombosis following rapamycin treatment was furthermore reported in lung cancer tumor xenografts [57]. 

Conditional cell depletion studies have partially confirmed the role of mTOR in endothelial cells and tumor angiogenesis. Specific ablation of tuberous sclerosis complex-1 (TSC1), a negative regulator of mTORC1, in endothelial cells resulted in the formation of lymphangiosarcoma characterized by sustained proliferation of endothelial cells [58]. Deletion of rictor, a component of mTORC2, in endothelial cells reduced VEGF-mediated endothelial cell proliferation. It also decreased the growth of tumor allografts, which was associated with diminished tumor angiogenesis [59]. The role of mTORC2 in VEGF signaling in endothelial cells was further confirmed using a phosphoproteomic approach [60]. Similarly, down-regulation of mTORC2 reduced prostaglandin E_2_-mediated endothelial cell responses and sprouting angiogenesis in vitro [61,62].

mTOR is ubiquitously expressed and its inhibition is not limited to endothelial cells. Accordingly, besides acting directly on endothelial cells, mTOR inhibitors influence angiogenesis by regulating the production of pro-angiogenic factors (Figure 2). Indeed, mTOR participates in the hypoxic tumor response by stabilizing hypoxia-inducible factor 1α and serves as a signaling intermediary in inflammation-mediated angiogenesis [63,64,65,66,67]. Consequently, mTOR inhibitors reduce the expression of VEGF [40,55].

**Figure 3:**
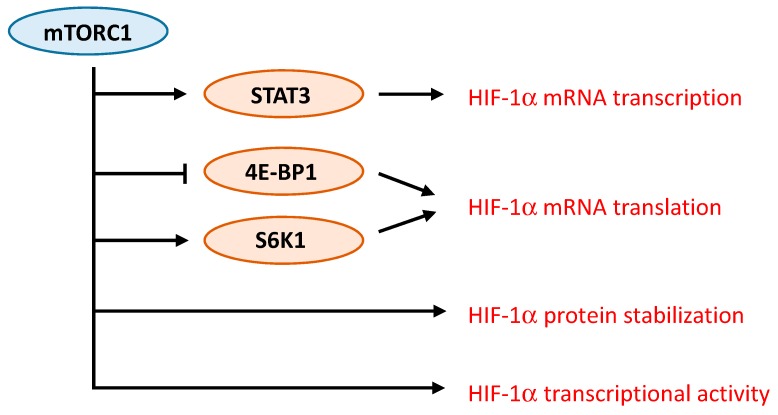
mTORC1 regulates HIF-1α signaling via different mechanisms. mTORC1 controls HIF-1α mRNA transcription and translation, HIF-1α protein stability and HIF-1α transcriptional activity.

Several studies have demonstrated the complex interaction between mTORC1 and HIF-1α. Different mechanisms responsible for the regulation of HIF-1α by mTORC1 have been proposed (Figure 3). For instance, in prostate cancer cells, rapamycin decreases protein levels of HIF-1α by interfering with processes that promote HIF-1α protein stabilization [63]. In contrast, activation of HER2 receptor in breast cancer cells increases HIF-1α synthesis via stimulation of HIF-1α mRNA translation in a rapamycin-sensitive manner [68]. Up-regulation of HIF-1α mRNA translation was further observed either as a result of increased cap-dependent translation following 4E-BP1 phosphorylation or via ribosomal protein S6 kinase-1 [69,70]. An additional mechanism involves the promotion of the transcriptional activity of HIF-1α by mTORC1 [71]. Finally, more recently, one study specifically addressed the mechanisms driven by mTORC1 that induce HIF-1α signaling [72]. The role of mTORC1 and its downstream substrates 4E-BP1 and S6K1 in regulating HIF-1α mRNA translation was confirmed. In addition, the regulation of HIF-1α mRNA transcription by mTORC1 was demonstrated, and appears to involve STAT3 [72].

Tumor angiogenesis is also influenced by tumor associated stromal cells [23]. In particular, tumor associated macrophages play an important role in shaping the angiogenic response in tumors, and can either sustain or, in contrast, repress angiogenesis [73,74]. Interestingly, mTOR activity in macrophages has been shown to be an important factor in promoting the ability of macrophages to stimulate angiogenesis [75]. Production of VEGF and interleukin-10 by human monocytes following lipopolysaccharide stimulation was significantly reduced when monocytes were treated with rapamycin, compared to untreated monocytes. Furthermore, in tumor xenograft models, depletion of macrophages is sufficient to inhibit the anti-angiogenic activity of rapamycin [75]. In contrast, infusion of monocytes with increased mTORC1 activity following genetic ablation of TSC2 results in increased tumor growth and angiogenesis in host mice bearing tumor xenografts [75]. Further evidence exists for a role of mTOR as a regulator of macrophages polarization [76].

Finally, besides sprouting angiogenesis, five other modes of vessel formation in tumors have been identified [77]. One is vascular mimicry, a process by which tumor cells acquire endothelial-like characteristics and line tumor vessels [25,78,79]. The occurrence of vascular mimicry is not frequent, but is nevertheless correlated with poor clinical outcome [80]. The observations that vascular mimicry correlates with mTOR expression and that rapamycin inhibits the expression of endothelial cell markers by tumor cells in vitro suggest that mTOR might further contribute to tumor blood supply by regulating vascular mimicry [81,82]. Additional studies are, however, needed to fully characterize the consequences of mTOR inhibition in this process.

## 3. Resistances to the Anti-Angiogenic Effects of mTOR Inhibitors

Several resistance mechanisms to anti-VEGF therapies have been characterized. For instance, the stimulation of tumor endothelial cells by other growth factors than VEGF has been identified [83,84]. As mentioned above, alternate modes of vascularization to sprouting angiogenesis are employed by tumors [85]. In addition to vascular mimicry, cancer cells can grow along pre-existing vessels by co-opting blood vessels [77]. Additionally, new blood vessels can be formed by intussusceptions, the splitting of pre-existing vessels to give rise to two daughter vessels.

In contrast to anti-VEGF treatments, resistances to the anti-angiogenic effects of mTOR inhibitors have barely been investigated. Nevertheless, emerging studies show that tumors are still able to maintain blood supply despite mTOR inhibition. In this context, lack of anti-angiogenic effects by mTOR inhibitors has been reported. Treatment of mice bearing human cervical carcinoma xenografts with rapamycin does not decrease intra-tumoral mean vessel density despite decreasing tumor growth [86]. It is of note that rapamycin treatment has no significant effect on plasma levels of VEGF in this study. Likewise, rapamycin fails to alter microvessel density in a transgenic mouse model of human epidermal growth factor receptor 2 (HER2)-positive breast cancer, even though mTORC1 inhibition in tumor endothelial cells was documented by immunohistochemistry [87]. Absence of anti-angiogenic effect has also been reported for the dual PI3K/mTOR inhibitor NVP-BEZ235, as well as for the mTORC1/mTORC2 inhibitor KU-0063794 [88,89]. In all these studies, tumor analysis was performed at the end of treatment. It is therefore not possible to differentiate whether tumor blood vessels were intrinsically resistant to rapamycin or, following an initial reduction of mean vessel density, alternate signaling pathways were engaged to compensate for the inhibition of mTOR and hence restore the formation of tumor blood vessels. Consistent with this latter hypothesis, it has been shown that treatment of endothelial cells with rapamycin increases the activity of mitogen-activated protein kinase (MAPK), which counteracts the anti-angiogenic efficacy of mTOR inhibitors [42]. Likewise, treatment of cultured endothelial cells with rapamycin increases the expression of the serine/threonine-protein kinase Pim-1, which reduces the anti-proliferative efficacy of rapamycin [90]. Interestingly, compensatory mechanisms of tumor blood supply have been detected upon inhibition of sprouting angiogenesis by rapalogs. In a rat model of hepatocellular carcinoma, electron microscopy analysis of tumors revealed that RAD001 reduces sprouting angiogenesis, and that under these circumstances, the main vascular growth mode is intussusception [91]. Hence, as for anti-VEGF therapies, resistance mechanisms to the anti-angiogenic activity of mTOR inhibitors exist and need to be thoroughly characterized. 

## 4. Combined Therapies to Increase the Anti-Angiogenic Efficacy of mTOR Inhibitors

Since the anti-cancer efficacy of mTOR inhibitors used as monotherapy was limited in cancer patients, pre-clinical studies have tested therapeutic approaches that combine mTOR inhibitors with other anti-cancer agents. Several reports have investigated the effects of such combined treatments on tumor angiogenesis. In this regard, the use of radiotherapy combined with mTOR inhibitors seems particularly interesting. Radiation increases mTORC1 activity in endothelial cells, suggesting that mTORC1 might counteract the effects of radiation [41]. Consistent with this hypothesis, rapalogs sensitize endothelial cells to radiation in culture by decreasing cell survival [41]. Combining rapalogs with radiation increases endothelial cell apoptosis as demonstrated by increased cleaved caspase-3 expression. Tubule formation by endothelial cells is inhibited to a greater extent by rapalogs in combination with radiation than by rapamycin or radiation alone [41]. Also, tumor growth of glioma allografts is significantly reduced by rapamycin in combination with radiation compared to either treatment alone. This effect is associated with reduced mean vessel density. Similar findings were reported in models of colon and pancreatic cancers, where disruption of VEGF production in cancer cells and VEGF-mediated signaling activation in endothelial cells induced by rapalogs were proposed as the underlying mechanisms [92]. Likewise, in sarcoma and non-small cell lung tumor xenografts, rapamycin treatment results in radio-sensitization, and reduction of tumor vessels is maximal under combined rapamycin-radiation treatment [93,94]. Interestingly, rapalogs further sensitize radio-resistant human oral squamous cell carcinoma tumor xenografts to fractionated radiation [95]. Compared to single treatments, combining rapalogs with fractionated radiation induces tumor endothelial cell apoptosis, which is associated with thrombus formation and tumor necrosis [95]. Dual PI3K/mTOR inhibitors demonstrated similar effects to rapalogs and radio-sensitized endothelial cells in vitro [96]. Based on these encouraging pre-clinical reports, phase I clinical trials combining rapalogs with radiation are performed.

Besides radiotherapy, combining mTOR inhibitors with chemotherapies shows additional anti-angiogenic activity. In the chick embryo chorioallantoic membrane, angiogenic response induced by neuroblastoma cells derived from cell lines or patients is maximally inhibited when rapamycin is combined with vinblastine [32]. Rapamycin also displays increased anti-angiogenic effects when administered with doxorubicin in a rat model of hepatoma [97].

Additionally, mTOR inhibitors have been tested in combination with anti-VEGF treatments. Co-administration of rapamycin and bevacizumab, a humanized recombinant monoclonal antibody that targets VEGF, was tested in mice bearing hepatocellular carcinoma tumor xenografts [98]. Combined treatments significantly reduced mean vessel density in tumor xenografts generated from six different cell lines. Importantly, the combination also decreased mean vessel density in a tumor xenograft that did not respond to single treatment, suggesting that the combination could overcome resistances to either treatment. The combination was also more efficient in decreasing VEGF levels [98]. Such combinations exhibited substantial activity and reasonable toxicity in advanced renal cell carcinoma and pancreatic neuroendocrine tumors in phase II trials [99,100]. In contrast, it was also reported that bevacizumab combined with CCI-779 did not provide any survival benefits compared to bevacizumab alone in advanced renal cell carcinoma [101].

Rapalogs were further tested with sorafenib and sunitinib, two small tyrosine kinase inhibitors that non-specifically target VEGF receptors. Formation of capillary tubes by endothelial cells was significantly more decreased by RAD001 in combination with sorafenib compared to single therapy [102]. Co-administration of sorafenib and RAD001 also decreased angiogenesis in osteosarcoma xenografts grown onto the chick embryo chorioallantoic membrane or in NOD/SCID mice. This effect was, however, not significantly different from treatment with sorafenib alone [102]. Other investigators reported that combined sorafenib/RAD001 neither decreased capillary tube formation nor significantly reduced endothelial cell proliferation compared to RAD001 alone [103]. However, combined treatment, when administered sequentially, significantly decreased endothelial cell sprouting from aortic rings compared to single treatment [103]. Absence of sprouting angiogenesis induced by combined sorafenib/RAD001 was further noted in a rat model of hepatocellular carcinoma [103]. Additional studies have revealed the potentiated anti-angiogenic effects of rapalogs combined with sunitinib. Association of rapamycin and sunitinib showed greater anti-angiogenic effects than rapamycin or sunitinib alone, both in vitro and in vivo [104]. The anti-angiogenic effect of RAD001 was also significantly increased in combination with TKI-258, another small tyrosine kinase inhibitor, in hepatocellular carcinoma tumor xenografts [105]. It is of note that a phase I clinical study revealed that sorafenib in combination with CCI-779 was associated with significant toxicity in metastatic melanoma patients [106]. Sunitinib combined with RAD001 in advanced renal cell carcinoma was also associated with toxicity and was only tolerated at attenuated doses.

As mentioned previously, treatment of endothelial cells with mTOR inhibitors results in increased MEK/MAPK pathway activity, which counteracts the anti-angiogenic efficacy of mTOR inhibitors [42]. Hence, combining MEK inhibitors with mTOR inhibitors provides greater anti-angiogenic effects, as evidenced in colon cancer and hepatocellular tumor xenografts [42,107]. Patients treated with such a therapeutic approach showed however non-negligible side effects that greatly limit its application in clinic [108].

Combining rapalogs with inhibitors of the insulin-like growth factor 1 receptor (IGF-1R) has also been tested. The rationale for such a combination is the observation that blocking IGF-1R abrogates rapamycin-mediated AKT activation in cancer cells [109]. Further evidence indicates that inhibitors of IGF-1R potentiate the anti-angiogenic efficacy of rapalogs by decreasing VEGF levels [110]. Clinical trials show contrasting results. While combining IGF1-R inhibitors with rapalogs in advanced sarcoma appears safe and provides anti-tumor activity [111,112,113], similar drug associations are no more effective than exemestane, an oral steroidal aromatase inhibitor, in estrogen receptor positive advanced breast cancer but exhibit more adverse effects [114].

Vascular disrupting agents target established tumor vasculature, which is distinct from anti-angiogenic agents that block neovascularization [115]. Hence, combining vascular disrupting agents with anti-angiogenic drugs is meaningful, and should result in increased anti-tumor activity. In this context, the effects of mTOR inhibitors combined with vascular disrupting agents have been tested [116,117]. In a three-dimensional spheroid sprouting assay, co-treatment of RAD001 with the vascular disrupting agent ASA404 significantly increased disruption of endothelial sprouts compared to single treatments. In vivo, ASA404 combined with RAD001 markedly increased tumor necrosis in a renal cell carcinoma model [116]. Similarly, NVP-BEZ235 combined with vascular-targeted photodynamic therapy showed a strong synergism characterized by increased endothelial cell apoptosis in vitro [117]. 

Finally, further pre-clinical studies showed that combined therapies can increase the anti-angiogenic effects of mTOR inhibitors. For example, co-administration of the histone deacetylase inhibitor LBH589 with rapamycin provides stronger anti-angiogenic effects in tumor xenografts compared to LBH589 or rapamycin alone. At a molecular level, this therapeutic strategy significantly reduces HIF-1α expression [118]. Likewise, methylnaltrexone, a peripheral-acting mu-opioid receptor antagonist, exerts a synergistic effect with CCI-779 or rapamycin on VEGF-induced endothelial cell proliferation and migration in cell culture and on angiogenesis in the matrigel plug assay [119]. Also, Toll-like receptor 9 agonist combined with RAD001 reduces VEGF production by renal cell carcinoma cells and impairs endothelial cell functions [120].

## 5. mTOR Inhibitors and Normalization of Tumor Vasculature

Due to excessive growth stimulation, tumor blood vessels display an aberrant morphology and poor functionality [121,122]. As a consequence, intra-tumoral fluid pressure is increased with areas of hypoxia that contribute to resistance to chemo- and radiotherapy. Hence, normalization of vascular abnormalities represents a therapeutic approach aiming to restore tumor blood perfusion and, thus, increased drug accessibility and reduced resistances mediated by hypoxia [123,124]. Tumor vessel normalization by anti-angiogenic drugs was initially observed with bevacizumab [125] and further investigated for mTOR inhibitors. Rapamycin reduces vessel permeability in a tumor xenograft model as evidenced by fluorescence tomography [126]. Similarly, rapamycin increases tumor perfusion and oxygenation in a model of rhabdomyosarcoma and potentiates the efficacy of radiotherapy [127]. Hence, rapamycin administration before irradiation to normalize the tumor vasculature represents a potential therapeutic strategy that needs to be precisely characterized. The observation that mTOR inhibitors sensitize various tumor xenografts to radiotherapy and chemotherapy further support such a therapeutic approach [93,128,129]. In addition, kinase inhibitors of mTOR provide similar effects on tumor vasculature normalization as rapalogs. NVP-BEZ235 decreases vascular permeability and accordingly intra-tumoral fluid pressure in a rat breast cancer model [50]. It further improves tumor oxygenation and response to radiotherapy [96]. Also, the mTORC1/mTORC2 inhibitor Palomid 529 inhibits VEGF-mediated increase of vascular permeability [54]. It is of note that the absence of effects of rapalogs on vascular permeability has also been reported, suggesting that the exact settings in which mTOR inhibitors induce vessel normalization have to be clearly identified [47,116].

## 6. mTOR Inhibitors and Tumor Endothelial Barrier

Tumor endothelium, by its unique position, regulates the trafficking of leukocytes into tumors by controlling the expression of adhesion molecules such as intercellular adhesion molecule-1 (ICAM-1) or vascular cell adhesion molecule-1 (VCAM-1) [130]. In addition, tumor endothelial cells are able to modify the activity of T lymphocytes as they express MHC major histocompatibility complex (MHC) class I and II as well as co-stimulatory and co-inhibitory molecules [131]. Hence, the tumor vasculature actively participates in the host tumor immune response. In the context of cancer, the endothelium is, however, most frequently anergic, failing to upregulate adhesion molecules and to properly recruit cytotoxic T cells. Moreover, tumor endothelium preferentially recruits T regulatory cells, further contributing to immune escape [132]. Furthermore, through the expression of Fas ligand, tumor endothelial cells are able to directly kill activated T lymphocytes [133]. Therefore, a therapeutic opportunity exists to shape tumor endothelium to promote an appropriate recruitment and activation of anti-tumor immune cells [134,135,136]. Emerging evidence suggests that mTOR inhibitors influence functions of endothelial cells that are relevant to host immune response. Expression of inhibitory molecules programmed death-ligand 1 (PD-L1) and programmed death-ligand 2 (PD-L2) is upregulated on endothelial cells both in vitro and in vivo upon rapamycin treatment [137]. Similarly, rapamycin reduces the expression of VCAM-1 on endothelial cells [138]. Hence, future experiments are needed to identify the effects of mTOR inhibitors on tumor endothelial barrier.

## 7. Biomarkers of Efficacy of mTOR Inhibitors 

The identification of biomarkers that predict sensitivity or resistance to mTOR inhibitors would be key to appropriately selecting patients likely to respond to these therapies. In this regard, molecular alterations of *PTEN*, *PI3K*, *KRAS* or Bcl-2 overexpression have been associated with either sensitivity or resistance to mTOR inhibitors [139,140,141]. The use of such biomarkers needs, however, to be validated in clinical trials. While these studies have mostly focused on one or few predefined molecules, the application of next generation sequencing represents a promising tool in the quest of biomarkers [142]. In fact, it has already been successfully applied in a patient with anaplastic thyroid cancer, who exhibited a near-complete response to the rapalog RAD001 for 18 months followed by disease progression [143]. Pre-treatment whole exome sequencing revealed the presence of a non sense mutation of *TSC2*, a negative regulator of mTORC1, resulting in overactivation of mTORC1. Similar analysis following tumor progression demonstrated *mTOR* mutations that render mTORC1 resistant to rapalogs. Likewise, activating mutations of mTOR were detected in a patient with metastatic urothelial carcinoma who had a fourteen months complete response to RAD001 [144]. It would be interesting to further test whether such mutations can be detected in liquid biopsies, which would provide an easy follow-up [145]. In addition, to date, no endothelial specific biomarker exists that specifically predicts anti-angiogenic response to mTOR inhibitors. Nevertheless, inhibition of endothelial Akt signaling has been identified as an important process responsible for the anti-angiogenic effects of rapalogs [146]. Indeed, high levels of endothelial Akt activity is associated with reduced anti-cancer effects of rapamycin.

## 8. Conclusions

mTOR inhibitors delay tumor progression in part by reducing tumor angiogenesis. This effect is, however, limited, as resistance mechanisms developed by cancer cells assure tumor blood supply despite mTOR inhibition. Thus, identification of these mechanisms is warranted to develop therapeutic strategies that may increase the efficacy of mTOR inhibitors. In this context, combinatory strategies have demonstrated interesting efficacy in pre-clinical studies. Translating these observations into clinical trials might, however, be associated with significant toxicity. In addition, mTOR inhibitors are able to normalize tumor blood vessels, suggesting a potential use as neo-adjuvant therapy prior to chemo- or radiotherapy. The precise settings in which mTOR inhibitors provide vasculature normalization effects need to be fully characterized. Finally, emerging evidence suggests that mTOR inhibitors might influence endothelial functions that participate in the tumor immune response. Future investigations are necessary to clarify this interrelation.
